# Every-Day Ailments

**Published:** 1887-04-16

**Authors:** 


### Till the Doctor Comes.

[Inquiries and Communications for this column should be addressed, "Physician," care of the Editor of The Hospital.]

### XV.—EVERY-DAY AILMENTS.

*Pediculi.*—Lice in the head. Best cured by cleanliness, washing with soft soap, and using remedies to kill the animals. Of these the most harmless is stavesacre ointment, which may be obtained at any chemist's. Some applications are dangerous, and others poisonous.

*Ringworm.*—Iodine paint is very useful when the ringworm is on the head, and lately, sulphur ointment has been highly spoken of; its advantage is that it is quite harmless. Cut the hair quite short on the patches before applying remedies. Be careful about using separate brushes and combs, towels, etc. When on the body it is much more easily cured; iodine paint is perhaps best. There are a large number of remedies recommended for this disease; a few more may be mentioned:—carbolic acid or creosote ointment, mercurial ointment, painting with blistering agents, and croton oil liniment, etc., but it is best to keep to the first-named remedies, and if the case does not then yield, call in medical advice.

*Vaccination.*—After vaccination no effect is noticed till about the third day, then there is a slight redness. On the fifth to the sixth day a small blister begins to form at each mark. On the eight day this blister attains its full size, and there is a red ring of inflammation around it. On the tenth or twelfth day the inflamed ring gradually appears, and the blister dries up. At the end of a fortnight a brown scab is formed, which falls off at the end of the third week. Care should be taken that the places do not get rubbed and irritated. Often there is a good deal of inflammation, and the glands in the armpit swell, but these symptoms will almost always disappear without any treatment. Fomenting with hot water or poppy infusion will relieve the pain if it is very severe.

*Vomiting.*—To relieve an urgent attack of vomiting till help arrives is often valuable time gained, and may prevent mischief. Warm drinks, etc., will usually be immediately rejected, but cold drinks taken in small quantities, and frequently, will tend greatly to allay the irritability of the stomach; effervescing drinks act in the same way. Thus, ice in small particles, iced milk and soda water, or iced brandy and soda water, will help to tide over the difficulty till skilled help can arrive. If the attack obviously depends on an undigested meal, an emetic of mustard and water may cure it at once. A mustard poultice over the stomach is also serviceable in most cases.

*Diarrhœa.*—In the diet of diarrhœa, farinaceous foods, as arrowroot, sago, etc., should be taken, and either cold or tepid, not hot. As a rule, brandy may be usually combined with them. Lime water and milk, or barley water, may be drunk, and rest in bed is advisable. It is impossible to prescribe any medicine without introducing opium or some drug of that character, which should never be used except under medical advice.

